# Immunomodulatory Natural Products in Cancer Organoid-Immune Co-Cultures: Bridging the Research Gap for Precision Immunotherapy

**DOI:** 10.3390/ijms26157247

**Published:** 2025-07-26

**Authors:** Chang-Eui Hong, Su-Yun Lyu

**Affiliations:** 1 College of Pharmacy, Sunchon National University, Suncheon 57922, Republic of Korea; gruni80@naver.com; 2 Smart Beautytech Research Institute, Sunchon National University, Suncheon 57922, Republic of Korea; 3 Research Institute of Life and Pharmaceutical Sciences, Sunchon National University, Suncheon 57922, Republic of Korea

**Keywords:** cancer organoids, natural products, immunomodulation, tumor microenvironment, precision medicine, 3D cell culture

## Abstract

Natural products demonstrate potent immunomodulatory properties through checkpoint modulation, macrophage polarization, and T cell/natural killer (NK) cell activation. While cancer organoid-immune co-culture platforms enable physiologically relevant modeling of tumor–immune interactions, systematic investigation of natural product immunomodulation in these systems remains entirely unexplored. We conducted a comprehensive literature analysis examining natural products tested in cancer organoids, immunomodulatory mechanisms from traditional models, technical advances in organoid-immune co-cultures, and standardization requirements for clinical translation. Our analysis reveals a critical research gap: no published studies have investigated natural product-mediated immunomodulation using organoid-immune co-culture systems. Even though compounds like curcumin, resveratrol, and medicinal mushroom polysaccharides show extensive immunomodulatory effects in two-dimensional (2D) cultures, and organoid technology achieves high clinical correlation for drug response prediction, all existing organoid studies focus exclusively on direct cytotoxicity. Technical challenges include compound stability, limited matrix penetration requiring substantially higher concentrations than 2D cultures, and maintaining functional immune populations in three-dimensional (3D) systems. The convergence of validated organoid-immune co-culture platforms, Food and Drug Administration (FDA) regulatory support through the Modernization Act 2.0, and extensive natural product knowledge creates unprecedented opportunities. Priority research directions include systematic screening of immunomodulatory natural products in organoid-immune co-cultures, development of 3D-optimized delivery systems, and clinical validation trials. Success requires moving beyond cytotoxicity-focused studies to investigate immunomodulatory mechanisms in physiologically relevant 3D systems, potentially unlocking new precision cancer immunotherapy approaches.

## 1. Introduction

Cancer immunotherapy has revolutionized oncology treatment, yet its clinical success remains limited, with response rates reaching approximately 20% by 2023 [1]. This modest efficacy, combined with significant adverse effects and high costs, necessitates innovative approaches to enhance therapeutic outcomes. Natural products, a vital source of anti-cancer drugs, represent a promising yet underexplored avenue for immunomodulation. Compounds such as curcumin, resveratrol, and medicinal mushroom polysaccharides demonstrate potent immunomodulatory properties through multiple mechanisms including checkpoint modulation, macrophage polarization, and immunogenic cell death induction [2,3,4,5].

The clinical translation of these immunomodulatory natural products has been severely hampered by inadequate preclinical models. Traditional two-dimensional cell cultures fail to recapitulate tumor architecture and drug responses [6] and are insufficient for modeling complex tumor immunobiology, lacking the cellular heterogeneity and three-dimensional structures essential for studying tumor–immune interactions [7,8]. Although animal models show poor predictive value for human toxicities in oncology trials [9], the species-specific immune differences are particularly problematic—mice and humans differ fundamentally in Toll-like receptor (TLR) expression, cytokine responses, and immune cell subset balances [10,11]. These limitations create a significant translational gap, contributing to the high failure rate of promising compounds in clinical trials.

Cancer organoids—three-dimensional structures derived from patient tumors—offer a transformative solution. Recent breakthroughs have enabled the co-culture of tumor organoids with autologous immune cells, creating physiologically relevant models that preserve tumor heterogeneity and immune interactions. Pioneering work by Dijkstra et al. demonstrated the generation of tumor-reactive T cells through co-culture of peripheral blood lymphocytes (PBL) with patient-derived tumor organoids [12]. Neal et al. developed an air-liquid interface (ALI) method that maintains diverse immune populations including T cells, B cells, natural killer (NK) cells, and macrophages while preserving native T-cell receptor repertoires [8]. These platforms achieve remarkable clinical correlation, with Votanopoulos et al. demonstrating strong concordance between organoid responses and patient outcomes in a subset of 15 metastatic cancer patients [13]. Larger studies in gastrointestinal cancers by Vlachogiannis et al. reported 100% sensitivity and 93% specificity for predicting patient treatment responses across 71 patients [14].

Although these technological advances, comprehensive exploration of natural product immunomodulation using organoid-immune co-culture systems remains in its infancy. While studies have demonstrated direct cytotoxic effects of natural compounds like curcumin [15] and resveratrol [16], the vast majority of existing work either uses simple organoid monocultures or focuses solely on direct anti-cancer effects, with organoid-immune co-culture investigations of natural products remaining absent from the literature. Notable examples include demonstrations of curcumin efficacy in patient-derived colorectal cancer organoids [15] and resveratrol effects in breast cancer organoids [16], yet thorough examination of immunomodulatory mechanisms—particularly involving immune cell co-cultures—is yet to be pursued.

The intersection of these fields is particularly timely given regulatory changes in 2022. The Food and Drug Administration (FDA) Modernization Act 2.0 now permits the utilization of organoid models as alternatives to animal testing, encouraging sponsors to advance these technologies [17]. This regulatory shift, combined with diverse opportunities for organoid-based investigations in precision medicine and immunotherapy development, creates significant potential for advancing cancer treatment [18].

To our knowledge, no published studies have comprehensively studied natural product-mediated immunomodulation using organoid-immune co-culture systems. Although numerous studies have tested natural compounds in cancer organoids for cytotoxicity and extensive literature exist on immunomodulatory mechanisms in traditional two-dimensional (2D) cultures, the key intersection—evaluating how natural products modulate tumor–immune interactions in three-dimensional (3D) organoid co-cultures— has not been investigated.

**Figure 1:**
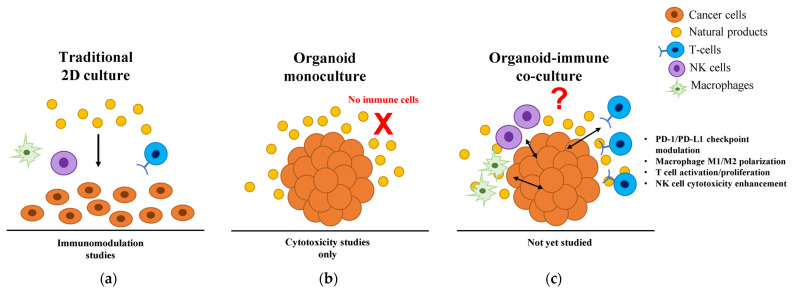
Current landscape of natural product research across different cancer model systems. (**a**) Traditional 2D culture systems enable immunomodulation studies but lack physiological relevance due to artificial cell arrangements and uniform drug distribution. (**b**) Current 3D organoid studies focus exclusively on direct cytotoxicity, with natural products tested only in monocultures lacking immune components (indicated by red X). (**c**) The proposed organoid-immune co-culture system would enable comprehensive evaluation of immunomodulatory mechanisms, including PD-1/PD-L1 checkpoint modulation, macrophage M1/M2 polarization, T cell activation/proliferation, and NK cell cytotoxicity enhancement. Even with the availability of validated co-culture technologies, no published studies have investigated natural product-mediated immunomodulation in these physiologically relevant 3D systems. 2D, two-dimensional; 3D, three-dimensional; M1, classically activated macrophage; M2, alternatively activated macrophage; NK, natural killer; PD-1, programmed cell death protein 1; PD-L1, programmed death-ligand 1.

This review comprehensively examines the current state and future potential of immunomodulatory natural products in cancer organoid models. We analyze (1) the limited but promising evidence of natural products tested in organoid systems, (2) methodological advances enabling organoid-immune co-culture studies, (3) insights from traditional immunomodulation research to identify promising candidates for organoid investigation, and (4) technical challenges and standardization requirements for clinical translation. By bridging these previously disparate fields, we aim to establish a framework for leveraging organoid technology to unlock the immunotherapeutic potential of natural products in precision oncology (Figure 1).

## 2. Background and Current Landscape

### 2.1. Fundamentals of Cancer Immunomodulation Pathways

The tumor microenvironment contains diverse immune cell populations whose functional states fundamentally determine therapeutic outcomes. Understanding these pathways is essential for developing effective immunomodulatory natural products.

CD8+ cytotoxic T-lymphocytes (CTLs) represent the primary effector cells for tumor elimination, but their function is often impaired through checkpoint molecules. The programmed cell death protein (PD)-1/PD-L1 axis serves as the dominant checkpoint, with PD-L1 expression on tumor cells binding to PD-1 on T cells to induce exhaustion characterized by reduced proliferation, cytokine production, and cytotoxicity [19,20,21]. Cytotoxic T-lymphocyte-associated protein 4 (CTLA-4) provides an earlier checkpoint by competing with CD28 for B7 ligands, preventing T cell activation [22,23]. Beyond exhaustion, T cells exist in multiple states including naive (CD45RA+CCR7+), central memory (CD45RO+CCR7+), effector memory (CD45RO+CCR7−), and terminally differentiated effector (CD45RA+CCR7−) populations, each with distinct capacities for tumor control [24,25,26].

Tumor-associated macrophages (TAMs) exhibit remarkable plasticity along the classically activated macrophages (M1)-alternatively activated macrophages (M2) polarization spectrum. M1, induced by interferon-gamma (IFN-γ) and lipopolysaccharide (LPS), express high levels of inducible nitric oxide synthase (iNOS), interleukin (IL)-12, and tumor necrosis factor-alpha (TNF-α), promoting anti-tumor immunity through direct cytotoxicity and T cell activation [27,28,29]. Conversely, M2, induced by IL-4/IL-13, express arginase-1, IL-10, and transforming growth factor-beta (TGF-β), supporting tumor growth through immunosuppression, angiogenesis, and tissue remodeling [30,31]. Yet, this binary classification oversimplifies TAM heterogeneity, as single-cell analyses reveal multiple intermediate states with mixed phenotypes that dynamically respond to microenvironmental cues [32,33,34].

NK cells provide rapid, antigen-independent tumor surveillance through activating receptors (NKG2D, NKp46, NKp30, NKp44) that recognize stress ligands on malignant cells [35,36]. NK cell cytotoxicity involves perforin/granzyme-mediated apoptosis and death receptor engagement (tumor necrosis factor-related apoptosis-inducing ligand (TRAIL), Fas ligand (FasL)) while activated NK cells produce IFN-γ to enhance adaptive immunity [37,38,39]. Nevertheless, tumors evade NK surveillance through ligand shedding, immunosuppressive cytokines (TGF-β, IL-10), and metabolic reprogramming that impairs NK cell fitness [40,41,42,43].

The tumor cytokine milieu orchestrates immune responses through complex feedback loops. Pro-inflammatory cytokines including IL-2, IL-12, and IFN-γ promote anti-tumor immunity by enhancing T cell proliferation, Th1 differentiation, and CTL function [43,44,45]. Conversely, immunosuppressive cytokines such as TGF-β, IL-10, and vascular endothelial growth factor (VEGF) create tolerogenic environments by inducing regulatory T cells (Tregs), myeloid-derived suppressor cells (MDSCs), and M2 macrophages [30,46,47]. IL-6 demonstrates context-dependent effects, promoting both inflammation and tumor progression through signal transducer and activator of transcription 3 (STAT3) signaling [48,49,50,51].

These interconnected pathways provide multiple targets for natural product intervention. Successful immunomodulation requires simultaneous engagement of multiple mechanisms—checkpoint inhibition alone may fail without concurrent macrophage reprogramming or cytokine modulation. Organoid-immune co-culture systems uniquely enable investigation of these complex interactions in physiologically relevant contexts, as detailed in subsequent sections.

### 2.2. Natural Products as Cancer Immunomodulators: Mechanisms Relevant to Organoid Applications

Natural products exhibit immunomodulatory effects through distinct mechanisms that are particularly relevant for organoid-based investigation. Four major categories demonstrate differential activities suitable for 3D culture systems.

Polysaccharides (predominantly β-glucans from medicinal mushrooms) activate pattern recognition receptors on immune cells within the tumor microenvironment. Lentinan, approved in Japan since the 1980s, demonstrates immunomodulatory effects through TLR4/Dectin-1 and spleen tyrosine kinase (Syk)-protein kinase C (PKC)-nuclear factor kappa B (NFκB) signaling pathways [52]. Polysaccharide-K (PSK), approved in 1977, acts as a novel TLR2 agonist that mediates tumor inhibition via stimulation of CD8+ T cells and NK cells [53,54]. Notwithstanding their therapeutic potential, these high molecular weight compounds (400–800 kDa for lentinan; 90–100 kDa for PSK) face unique challenges in penetrating dense tumor matrices.

Polyphenols including curcumin, resveratrol, and epigallocatechin-3-gallate (EGCG) modulate multiple immunological pathways simultaneously. These compounds suppress PD-L1 expression through diverse mechanisms—curcumin via CSN5 inhibition leading to PD-L1 destabilization [55] and STAT3 pathway blockade [56], EGCG through JAK2/STAT1 inhibition [57], and resveratrol by disrupting PD-L1 glycosylation and dimerization [58]. Though traditionally known for anti-inflammatory effects that promote M2 macrophage polarization [59,60], emerging evidence suggests context-dependent immunomodulation where certain formulations may enhance anti-tumor immunity [61,62]. This apparent paradox stems from an oversimplified M1/M2 dichotomy, as recent transcriptome-based network analysis of 299 macrophage samples demonstrates that macrophage activation exists along a spectrum with at least nine distinct activation programs rather than binary states [63]. In this context, polyphenols induce concentration-dependent and context-specific macrophage responses—for example, resveratrol demonstrates biphasic effects where moderate concentrations (20 μM) downregulate M2 markers (MRC1, CCL24, Chi3l3) while higher concentrations favor M2 polarization through phosphatidylinositol 3-kinase (PI3K)/Akt and AMP-activated protein kinase (AMPK) pathways [64]. This complexity, combined with evidence that M2-like tumor-associated macrophages can be reprogrammed to anti-tumor phenotypes [65], highlights how polyphenol immunomodulation cannot be adequately studied in simplified 2D systems. Organoid-immune co-culture platforms offer the unique opportunity to investigate how tumor microenvironmental factors, drug concentration, and exposure timing determine whether polyphenol-induced macrophage modulation ultimately supports or inhibits tumor growth.

Complex extracts demonstrate immunomodulatory effects through their bioactive components. *Panax ginseng* berry polysaccharides (GBPP) enhance immune responses by activating macrophages and NK cells, achieving significant anti-cancer effects through cytokine production (IL-6, IL-12, and TNF-α) [66]. *Ganoderma lucidum* contains both polysaccharides and triterpenoids, with organic solvent extracts showing direct cytotoxicity through IL-6/IL-8 suppression [67], commercial extracts inhibiting tumor growth via mechanistic target of rapamycin (mTOR) pathway modulation [68], and multiple immunomodulatory mechanisms [69]. These findings align well with organoid platforms that enable systematic evaluation of immune-tumor interactions [70].

**Figure 2:**
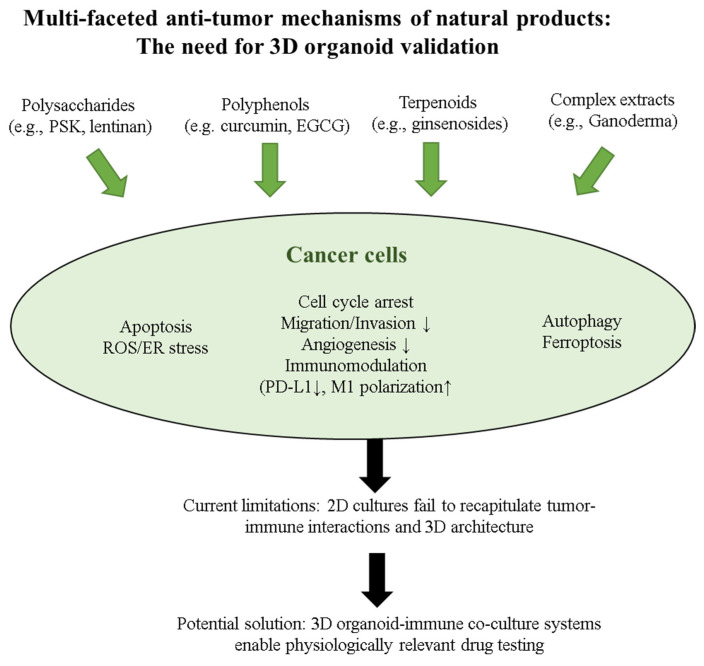
Multi-faceted anti-tumor mechanisms of natural products requiring validation in 3D organoid-immune co-culture systems. Natural products from four major categories (polysaccharides, polyphenols, terpenoids, and complex extracts) exhibit diverse anti-tumor activities through multiple mechanisms: direct cytotoxicity (apoptosis, cell cycle arrest, autophagy, ferroptosis), oxidative stress induction (ROS/ER stress), immunomodulation (PD-L1 downregulation, M1 macrophage polarization), and tumor microenvironment regulation (migration/invasion and angiogenesis inhibition). While these mechanisms have been extensively characterized in 2D cultures, current models fail to recapitulate tumor–immune interactions and 3D architecture. The implementation of 3D organoid-immune co-culture systems represents a crucial advancement for physiologically relevant drug testing and precision cancer therapy development. 2D, two-dimensional; 3D, three-dimensional; EGCG, epigallocatechin-3-gallate; ER, endoplasmic reticulum; M1, classically activated macrophage; PD-L1, programmed death-ligand 1; PSK, polysaccharide-K; ROS, reactive oxygen species. Symbols: ↓ indicates decreased or downregulated expression, ↑ indicates increased or upregulated expression.

The focus on key immunological targets—checkpoint molecules, cytokine networks, macrophage polarization, and T cell/NK cell activation—provides clear endpoints for organoid-based assessment [71,72]. Importantly, these mechanisms require intact tumor–immune interactions that only 3D systems can adequately model, as demonstrated in patient-derived organoid (PDO) co-culture studies achieving 33–50% success rates for generating tumor-reactive T cells [12]. The diverse anti-tumor mechanisms of natural products—encompassing direct cytotoxicity, immunomodulation, and tumor microenvironment regulation—underscore their therapeutic potential that demands validation in physiologically relevant 3D systems (Figure 2).

### 2.3. Limitations of Current Models Driving the Need for Organoid Platforms

In spite of their limitations, two-dimensional cell culture models remain a standard platform for initial natural product screening and mechanistic studies [73,74]. These models enabled the initial identification of key immunomodulatory compounds, including characterization of curcumin’s NF-κB inhibitory effects [75,76,77], resveratrol’s SIRT1 activation [78,79,80,81], and EGCG’s effects on T cell differentiation [82,83,84]. The simplicity, cost-effectiveness, and high-throughput capability of 2D cultures facilitated screening of thousands of natural compounds, establishing fundamental structure-activity relationships that guide current research [85,86,87,88]. Moreover, mechanistic insights into checkpoint modulation, cytokine signaling, and immune cell activation pathways by natural products have been extensively studied [89,90,91,92].

Nevertheless, two-dimensional cultures fundamentally alter immune-related responses. Comparative studies reveal that 3D spheroids showed relatively lower activities in the AKT-mTOR-S6K signaling pathway compared to cells cultured in two dimensions, with significantly altered drug sensitivities [6]. The transition from 2D to 3D culture significantly impacts cell proliferation, differentiation, and survival under some circumstances, resulting in cellular behaviors that differ from those in vivo [93]. For immunomodulatory compounds, this discrepancy is amplified by the absence of physiological cell–cell interactions, gradient formation, and mechanical forces.

Drug resistance patterns differ markedly between culture dimensions. Triple-negative breast cancer cells demonstrated significantly higher half-maximal inhibitory concentration (IC50) values for epirubicin, cisplatin, and docetaxel in 3D cultures compared to 2D across 13 tested cell lines [94]. Natural products show similar patterns—marine-derived *Caulerpa sertularioides* extract exhibited IC50 values of 80.28 μg/mL in 2D versus 530 μg/mL in 3D cultures after 24 h, representing a 6.6-fold increase in resistance [95], while crocin treatment showed IC50 values of 3.68 mg/mL in 2D versus 10.12 mg/mL in 3D cultures, representing a 2.77-fold increase that further elevated to 4.34-fold by 72 h [96]. These findings underscore the inadequacy of monolayer systems for predicting therapeutic responses, failing to achieve clinical approval [97].

Animal models have made indispensable contributions to natural product immunology research. Murine models enabled the discovery of immunomodulatory mechanisms for numerous compounds now in clinical development, including demonstration of PSK’s TLR2-mediated immune activation [53,54,98,99] and lentinan’s enhancement of anti-tumor immunity through macrophage activation [100,101,102,103]. These models provided essential pharmacokinetic and safety data that facilitated the clinical approval of mushroom polysaccharides in Japan [101,104,105]. Furthermore, syngeneic tumor models allowed investigation of complex tumor–immune interactions, revealing how natural products modulate the tumor microenvironment through effects on MDSCs, Tregs, and TAMs [91,106,107]. Mechanistic studies using knockout mice have elucidated specific molecular targets and signaling pathways essential for natural product activity [108,109,110,111].

In contrast, animal models present species-specific immune barriers that have led to numerous translational failures in natural product development. Mestas and Hughes cataloged fundamental immunological differences between mice and humans that directly impact natural product efficacy [10]. These include (1) divergent TLR expression patterns—TLR10 is completely absent (pseudogene) in mice while widely expressed in humans, and TLR9 is expressed on all murine myeloid cells but restricted to B cells and plasmacytoid dendritic cells (DCs) in humans; (2) contrasting cytokine responses—human Th1 cells produce IL-10 while murine Th1 cells do not, and IL-8 (CXCL8), crucial for human neutrophil recruitment, is entirely absent in mice; (3) reversed immune cell subset balances—human blood contains 50–70% neutrophils and 30–50% lymphocytes, while mouse blood shows 10–25% neutrophils and 75–90% lymphocytes, alongside completely different NK cell receptor families (Ly49 in mice vs. killer cell immunoglobulin-like receptor (KIR) in humans) [10].

These profound differences have resulted in significant translational failures for natural products. Curcumin demonstrated potent anti-inflammatory effects in murine models but showed minimal bioavailability and efficacy in human trials [112,113,114]. Resveratrol’s robust immunomodulatory activity in mice failed to translate to humans due to species-specific differences in metabolism and SIRT1 pathway activation [115,116,117]. Most strikingly, TLR9 agonists like cytosine-phosphate-guanine (CpG) oligonucleotides that showed promising anti-tumor immunity in mice caused severe adverse events in human trials due to the broader TLR9 expression on human immune cells [118,119,120,121,122].

PDOs offer a compelling alternative that circumvents these species-specific barriers. PDOs maintain patient-specific genetic backgrounds and tissue architecture while enabling co-culture with autologous human immune cells, thereby preserving human-specific TLR expression patterns, cytokine networks, and immune cell ratios [12,123,124,125,126]. Unlike murine models, PDO-immune co-cultures accurately recapitulate human IL-8/CXCR1 signaling, human-specific Th1 IL-10 production, and authentic human neutrophil-lymphocyte interactions, providing more predictive platforms for natural product immunomodulator screening [122,127,128,129].

Seok et al. demonstrated that genomic responses in mouse models poorly mimic human inflammatory diseases, with key pathways showing opposite directional changes between species [11]. Meta-analyses show that animal models achieve only 0.65 positive predictive value for human toxicities, likely lower for complex immunomodulatory effects [9].

Pharmacokinetic disparities further complicate translation. Natural products face unique challenges: curcumin achieves only nanomolar plasma concentrations (22–41 ng/mL) despite 8 g daily dosing [130], while oral polysaccharides show extremely poor oral bioavailability of 0.5–4.9% depending on molecular structure [131]. These issues cannot be adequately modeled in systems lacking human-specific metabolism and immune components.

### 2.4. Organoid Platforms: Bridging the Translational Gap

PDOs demonstrate remarkable potential as predictive models for drug response. In a landmark study of metastatic gastrointestinal cancers by Vlachogiannis et al. (n = 71), organoid drug sensitivity testing achieved 100% sensitivity and 93% specificity in predicting treatment responses in 15 patients from their larger cohort (n = 71), establishing a robust foundation for precision medicine applications [14]. This predictive capability has been further validated in cancer-specific contexts, where organoids maintain essential tumor characteristics essential for accurate modeling. Pancreatic cancer organoids have proven particularly valuable, as they recapitulate the mutational spectrum and transcriptional subtypes of primary tumors through combined genomic, transcriptomic, and therapeutic profiling, enabling comprehensive assessment of treatment vulnerabilities [132]. Nonetheless, the clinical translation of organoid-based predictions reveals important drug-specific variations in accuracy. Whereas colorectal cancer organoids demonstrate >80% accuracy for irinotecan-based therapies, they notably failed to predict outcome for 5-fluorouracil plus oxaliplatin combinations, highlighting the need for therapy-specific validation of organoid predictive models [133]. These findings underscore both the promise and limitations of organoid technology, emphasizing that successful clinical implementation requires careful consideration of cancer type, treatment regimen, and individual drug mechanisms when interpreting organoid-based predictions for patient care.

Immune integration breakthroughs have transformed organoid capabilities. The development of ALI cultures by Neal et al. enables maintenance of endogenous tumor-infiltrating lymphocytes, macrophages, and other immune populations for over 10 days with IL-2 supplementation [8]. Recent innovations include autologous T cell generation achieving 33–50% success rates in colorectal and lung cancers [134,135], generation of tumor-reactive T cells by co-culture of PBL and tumor organoids, sustained cytokine production matching in vivo patterns, and functional assessment of checkpoint blockade responses [136,137,138].

Technical standardization is rapidly maturing. Korean and Chinese organoid initiatives have published comprehensive guidelines [139,140,141,142,143,144], while automated platforms demonstrate excellent reproducibility with average coefficient of variation (CV) of 3.56% for drug screening [145]. These advances address previous reproducibility concerns while enabling high-throughput natural product screening.

Clinical correlation validates the platform’s utility. A systematic review of organoid studies demonstrate 81% sensitivity and 74% specificity for predicting patient responses in pooled analyses, with subset analyses of studies containing ≥5 responders and non-responders achieving 84% sensitivity and 81% specificity [146]. Patient-derived ovarian cancer organoids display inter- and intrapatient drug response heterogeneity, with PDO responses to carboplatin/paclitaxel showing statistically significant correlation (*p* < 0.01) with clinical outcomes [147]. Combined with 2–3 week turnaround times, this positions organoid-guided natural product selection as a viable clinical tool.

The combination of preserved architecture, functional immune integration, improving standardization, and clinical validation creates a unique opportunity to systematically evaluate immunomodulatory natural products in human-relevant systems.

## 3. Natural Products in Cancer Organoid Systems: Current Evidence

The intersection of natural product research and cancer organoid technology remains remarkably limited. Our comprehensive literature analysis reveals a major gap: even as natural compounds have been tested in cancer organoids and their immunomodulatory properties have been extensively studied in conventional systems, the investigation of natural product-mediated immunomodulation using organoid-immune co-culture platforms is yet to be examined. Here we synthesize available evidence from two distinct categories: (1) natural products tested in cancer organoids for cytotoxic effects, and (2) immunomodulatory mechanisms demonstrated in traditional 2D cultures or animal models that require translation to organoid platforms (Table 1).

Important note: Unless otherwise specified, all natural product studies in organoids discussed below evaluated direct cytotoxicity only, without immune cell involvement. This absence of immunomodulatory investigation in 3D co-culture systems represents the fundamental gap this review addresses.

### 3.1. Direct Evidence from Organoid Studies

#### 3.1.1. Polyphenolic Compounds in PDOs

The landscape of polyphenolic compound testing in PDOs is surprisingly sparse. Even though the extensive literature on natural product anti-cancer activities in traditional models, comprehensive assessment in PDO systems is still at an early stage, with existing studies now expanding beyond cytotoxicity to include diverse endpoints such as signaling pathways, metabolomics, and stemness markers (Table 2).

Luteolin represents one of the few comprehensively studied flavonoids in PDOs. Hao et al. [150] tested luteolin in 11 patient-derived gastric cancer organoids, revealing an IC50 of 27.19 μM for direct cytotoxic effects, which demonstrated superior efficacy compared to carboplatin (IC50: 37.87 μM) but was comparable to norcantharidin (IC50: 23.9 μM). The compound exhibited differential sensitivity patterns across patient samples, highlighting the potential for personalized therapeutic approaches. Other polyphenolic compounds remain largely unexplored in true PDO systems. While numerous studies have investigated compounds such as curcumin, resveratrol, quercetin, and EGCG in cell line-based 3D cultures or spheroids, these models lack the patient-specific heterogeneity and tissue architecture of authentic PDOs. Importantly, EGCG has demonstrated extensive immunomodulatory effects in conventional 2D cancer models, including modulation of cytotoxic lymphocytes, dendritic cells, myeloid-derived suppressor cells, and regulatory T cells [156], yet PDO-immune co-culture studies have not been conducted.

The scarcity of polyphenol PDO studies is further highlighted by recent efforts in related natural product research. Studies have explored other natural compounds in organoid systems, such as Chaga mushroom (*Inonotus obliquus*) extracts in canine bladder cancer organoids [155], suggesting the technical feasibility of natural product screening in organoid platforms while underscoring the gap in polyphenolic compound investigation.

The extreme paucity of polyphenolic compound studies in PDOs represents a significant missed opportunity in personalized cancer medicine. PDOs have emerged as powerful platforms for precision oncology, offering multiple advantages for drug testing and therapeutic prediction [157]. These models maintain patient-specific tumor characteristics while enabling high-throughput screening and mechanistic studies in a controlled environment [158]. The integration of validated PDO technology with the therapeutic potential of polyphenolic compounds presents an unexplored frontier for developing personalized natural product-based cancer therapies.

#### 3.1.2. Polysaccharides and Complex Extracts

Although extensive evidence of anti-cancer activity in traditional systems, polysaccharide evaluation in true cancer organoid platforms remains remarkably limited, with existing studies focusing exclusively on cytotoxic effects. Natural product screening in PDOs is still an emerging field [159], with organoid research only gaining significant momentum after 2015 while most polysaccharide cancer studies predate this technological shift. Modified citrus pectin (MCP) shows promising synergy with conventional therapies in 3D systems, though tested only for direct anti-cancer effects. Pectasol-C MCP (0.025%) combined with paclitaxel in SKOV-3 ovarian cancer spheroids demonstrated significant viability reduction through galectin-3 inhibition and STAT3 pathway disruption [160]. Still, this represents spheroid rather than true organoid evaluation, and immunomodulatory potential has not been evaluated.

Chaga mushroom extract evaluation in canine bladder cancer organoids represents the most comprehensive 3D study for medicinal mushrooms, assessing cytotoxicity and cell cycle arrest only. The study achieved dose-dependent viability reduction with 25–100 μg/mL inducing G0/G1 arrest after 72 h treatment [155]. In spite of mushroom polysaccharides’ well-documented immunostimulatory effects in 2D systems, this veterinary model focused solely on direct anti-cancer mechanisms.

Luteolin from plant sources demonstrates exceptional cytotoxic activity in gastric cancer organoids (IC50: 27.2 μM), outperforming carboplatin while maintaining selectivity for malignant cells [150]. PDO responses correlated with clinical outcomes in 11/12 cases, validating the platform’s predictive value for cytotoxic agents—without any immunomodulatory assessment.

The limited number of organoid studies, particularly for immunomodulatory assessment, likely reflects technical challenges rather than a lack of therapeutic potential. High molecular weight polysaccharides face unique barriers including limited penetration through Matrigel, as Phan et al. demonstrated that “Matrigel does not form representative barriers to ovarian cancer cells in either 2D or 3D culture systems” [161], concentration gradients from periphery to center demonstrated through matrix-assisted laser desorption/ionization (MALDI) imaging and microfluidic studies [162,163], and susceptibility to enzymatic degradation in long-term culture conditions. Although Berlemont comprehensively analyzed fungal polysaccharide-degrading enzymes [164], the specific impact on organoid cultures is still unknown. Even with extensive preclinical evidence of immunomodulatory activity in simpler model systems, marine-derived fucoidan (molecular weight (Mw) = 469 kDa) and mushroom β-glucans—with PSK (90–100 kilodalton (kDa)) approved as an oral adjuvant and lentinan (400–800 kDa) as an intravenous formulation in Japan, highlighting how delivery route impacts clinical translation specifically for their immunostimulatory effects [52,165]—have never been evaluated in organoid-immune co-culture platforms. *Dendrobium officinale* polysaccharides ranging from 24.89 kDa to 1224.54 kDa have shown anti-cancer effects [166], highlighting the diverse molecular weight range of bioactive polysaccharides.

Systematic screening of natural product libraries in organoid platforms represents an unmet need. Even though computational approaches have identified promising candidates from traditional medicine databases, with 5278 anti-cancer compounds predicted from traditional Chinese medicine (TCM) database [167], and comprehensive natural product libraries have been established [85], their application to organoid-based screening is not yet fully established. The successful application of automated platforms achieving coefficient of variation of 3.56% for size distribution and <9% for cellular composition [145] combined with recent standardization guidelines [138,139,140,141,142,143,144], now enables high-throughput polysaccharide discovery. This synergy between technical capabilities and regulatory support creates exceptional opportunities for advancing these historically important but understudied compounds.

### 3.2. Evidence of Immunomodulation from 2D and Animal Models

Although organoid-immune co-culture studies are lacking, extensive studies in 2D cultures and animal models demonstrate significant immunomodulatory potential of natural products. These findings, though not yet validated in organoid platforms, provide a promising rationale and roadmap for future organoid-based investigations. Here we review key immunomodulatory mechanisms that warrant immediate translation to organoid-immune co-culture systems.

#### 3.2.1. Checkpoint Modulation Capabilities

Natural products demonstrate potent checkpoint modulation in traditional systems, yet these mechanisms need validation in organoid-immune co-cultures. Resveratrol inhibits PD-L1 glycosylation in 2D cancer cell cultures, preventing proper membrane localization through endoplasmic reticulum retention [58]. This mechanism appears particularly relevant for organoid-immune co-cultures where spatial organization and cell–cell interactions are preserved.

Multiple compounds demonstrate checkpoint modulation requiring prompt organoid validation:

- Curcumin: Enhances PD-L1 ubiquitination via CSN5 downregulation in 2D systems [55]
- Ginsenoside Rh2: Augments anti-PD-L1 immunotherapy by reinvigorating CD8+ T cells through increasing intratumoral CXCL10 signaling in xenograft models [168]
- Astragalus polysaccharides: Suppress PD-L1 via AKT/mTOR/p70S6K signaling in cancer cell lines [169]
- EGCG: Inhibits PD-L1/PD-L2 expression through JAK-STAT signaling [170] and separately suppresses PD-1 expression via NF-κB pathway modulation [171]

These mechanisms, demonstrated only in simplified 2D cultures or immunodeficient mouse models, urgently require validation in human-relevant organoid-immune co-culture systems.

Translation to organoid platforms: These checkpoint modulation mechanisms represent priority targets for validation in organoid-immune co-cultures. Future studies should leverage established co-culture protocols [134] while addressing the unique challenges of natural product delivery in 3D systems, including optimization of compound concentrations and exposure timing.

#### 3.2.2. Macrophage Polarization in 3D Contexts

Limited organoid-macrophage studies provide proof of concept, though none have evaluated natural product effects. Zhang et al. demonstrated that transplanted intestinal organoids attenuate ischemia/reperfusion injury through endogenous L-malic acid production, which induces M2 macrophage polarization via suppressor of cytokine signaling 2 (SOCS2)-dependent pathways in vivo [172]. Though this represents an in vivo transplantation model rather than direct natural product testing, it confirms organoids can modulate macrophage phenotypes—a capability yet to be exploited for natural product screening.

Traditional compounds show macrophage modulation in 2D systems that require investigation in organoid systems. Contrary to promoting M1 polarization, both curcumin and EGCG actually suppress M1 and enhance M2 polarization—curcumin reduces inflammatory cytokines while increasing anti-inflammatory markers like IL-10 and arginase-1 (Arg-1) through NF-κB inhibition [173,174], and EGCG suppresses M1 markers (iNOS, TNF-α) while upregulating M2 markers (Arg-1, Krüppel-like factor 4 (KLF4)) [175]. Various polysaccharides from medicinal mushrooms promote M1 polarization through TLR4 activation, as demonstrated by *Ganoderma lucidum* polysaccharides inducing pro-inflammatory cytokines via TLR4-myeloid differentiation primary response 88 (MyD88) signaling [176,177]. These opposing effects—some compounds promoting anti-tumor M1 polarization while others induce potentially tumor-supportive M2 phenotypes—highlight the vital need for organoid-based evaluation in physiologically relevant tumor microenvironments.

Organoid applications: The successful maintenance of endogenous immune populations in organoid cultures, particularly through ALI platforms [8], provides an opportunity to investigate natural product-mediated macrophage polarization in physiologically relevant contexts. Adaptation of existing organoid-immune co-culture methods [134] will be essential for translating these immunomodulatory mechanisms.

### 3.3. Cancer Type-Specific Patterns Emerging from Limited Data

Early evidence suggests natural product cytotoxic responses in organoids follow cancer-specific patterns, though immunomodulatory effects have not been studied across all cancer types:

Gastrointestinal cancers demonstrated consistent cytotoxic responses to natural products. Colorectal cancer organoids show varying establishment rates, with Vlachogiannis et al. reporting 70% success rate [14] and gastric cancer showing 66.6% success rate in meta-analysis [148]. Natural products demonstrate consistent cytotoxic responses, with curcumin showing IC50 values of 20–50 μM across colorectal cancer organoids [151], and luteolin achieving IC50 of 27.19 μM in gastric cancer organoids [150]. Regardless of the well-established role of immune dysfunction in GI cancers and extensive evidence of natural product immunomodulation in 2D systems, immune-mediated mechanisms in GI cancer organoid co-cultures have not been investigated.

Breast cancer organoids showed high sensitivity to polyphenol cytotoxicity. Resveratrol induced > 50% cell death in 79.2% (19/24) of treated organoids [16], while quercetin demonstrated chemosensitizing effects with IC50 values less than 22 μM, enhancing sensitivity to conventional chemotherapy through PTEN/Akt and ERK1/2 pathway modulation [153]. Given breast cancer’s known responsiveness to immunotherapy and natural products’ documented effects on tumor-associated macrophages and T cells in 2D models, organoid-immune co-culture studies represent an unexplored opportunity.

Lung cancer models revealed promising cytotoxic responses to marine-derived natural products. Manoalide at 10–15 μM promoted epidermal growth factor receptor-tyrosine kinase inhibitor (EGFR-TKI) sensitivity through Kirsten rat sarcoma viral oncogene homolog-extracellular signal-regulated kinase (KRAS-ERK) pathway inhibition and ferroptosis induction [154]. On the other hand, considering lung cancer’s high mutational burden and potential for immune recognition, the lack of studies examining how natural products might enhance anti-tumor immunity in lung cancer organoids is particularly concerning.

Pancreatic cancer organoids have been successfully established with 75% efficiency from 101 patients [132], providing validated platforms for drug testing. Though direct natural product cytotoxicity studies remain limited, the aggressive immunosuppressive microenvironment of pancreatic cancer makes it an ideal candidate for testing immunomodulatory natural products—yet immunomodulatory natural product studies are notably lacking.

The pattern is clear: across all major cancer types, natural product research in organoids has focused exclusively on direct cytotoxicity, overlooking their immunomodulatory potential despite convincing evidence from other model systems.

### 3.4. Technical Insights from Early Adopters

Pioneering studies reveal important considerations for natural product testing in organoids, though all insights derive from cytotoxicity assessments rather than immunomodulatory investigations.

Concentration dynamics: Organoids consistently require higher concentrations than 2D cultures for cytotoxic effects. Hundsberger et al. demonstrated that quercetin required 31-fold higher concentrations in 3D melanoma spheroids (12.5 μM) compared to 2D cultures (0.4 μM) for caspase 3 activation [178]. This dramatic difference underscores the importance of concentration optimization when transitioning from traditional screening to organoid platforms [179,180]. For immunomodulation studies, this poses a serious challenge: the substantially higher concentrations required for tumor effects in 3D may compromise immune cell viability, creating a narrow therapeutic window where anti-tumor effects must be balanced against maintaining functional immune populations.

Temporal patterns: Drug responses in organoids follow time-dependent phases for cytotoxicity. Optical metabolic imaging demonstrates treatment responses within 24 h in pancreatic cancer organoids [181], while Chen et al. showed that resveratrol requires 96 h exposure for full anti-cancer effects through ferroptosis induction [152]. These findings align with optimization studies demonstrating optimal timing for patient response correlation [157,182].

Formulation requirements: Hydrophobic compounds necessitate specialized delivery systems. Zou et al. demonstrated that liposomal paclitaxel formulations showed superior efficacy compared to albumin-bound forms in gastric cancer organoids, achieving 90% organoid establishment rate [183]. The additional complexity of maintaining functional immune cells while delivering natural products presents an unexplored technical challenge.

Heterogeneity assessment: Single-organoid analysis reveals cytotoxic response variations. Tebon et al. developed high-speed live cell interferometry for single-organoid drug screening [184], while Sharick et al. demonstrated metabolic heterogeneity both between primary sites and within organoid populations [181]. For immunomodulatory assessment, this heterogeneity would be compounded by immune cell variability [159,185].

The lack of direct evidence paradoxically highlights the opportunity. With validated organoid-immune co-culture platforms expanding rapidly as reviewed by Papp et al. [7] and Wang et al. [186], combined with extensive knowledge of natural product immunomodulation from traditional systems used since antiquity [187], the field stands poised for rapid advancement through methodical study.

## 4. Technical Challenges and Standardization

Given that the absence of natural product immunomodulation studies in organoid-immune co-cultures presents a clear opportunity, several technical challenges must be addressed to enable rigorous analysis. These challenges are amplified when considering the unique physicochemical properties of natural products combined with the complexity of maintaining functional immune populations in 3D culture systems.

### 4.1. Natural Product-Specific Challenges in Organoid Systems

#### 4.1.1. Formulation and Bioavailability Barriers

Natural products face unique physicochemical challenges in 3D organoid matrices that significantly impact their immunomodulatory potential. Hydrophobic compounds exemplify these issues: curcumin’s aqueous solubility (approximately 11 ng/mL at pH 5.0) results in heterogeneous Matrigel distribution, severely impeding its gastrointestinal uptake [188,189]. VEGF diffusion studies in Matrigel demonstrate the complexity of molecular transport, with coefficients ranging from 0.24 mm^2^/day (theoretical) to 24 mm^2^/day (empirical measurements), highlighting the variability in 3D matrix penetration [190].

Stability compounds the challenge. Curcumin exhibits pH-dependent stability, retaining only 53–62% at physiological pH (7.0–8.0) after 1 month at 37 °C [191]. The presence of 10% fetal calf serum (FCS) improves stability (<20% decomposition within 1 h), though 50% degradation still occurs after 8 h even with serum protection [192]. pH-dependent stability is particularly relevant: curcumin retains > 85% activity at pH < 7 after 1 month at 37 °C, but only 53–62% at physiological pH 7.0–8.0 [191]. Natural products also demonstrate extensive binding to human serum albumin, which can enhance their stability and bioavailability through protective protein interactions [193]. This pH sensitivity presents unique challenges for immunomodulation studies. Immune cells require physiological pH for optimal function, yet at this pH curcumin shows significant degradation. This compromise sustained immunomodulatory effects during critical immune activation windows, necessitating either continuous compound replenishment or protective formulations that maintain both compound stability and immune cell viability.

#### 4.1.2. Delivery System Innovations

Recent advances demonstrate quantifiable improvements. At the same time, these delivery systems must consider immune cell compatibility. Different formulation strategies may influence interactions with immune cells in the tumor microenvironment, potentially affecting the intended immunomodulatory outcomes. Polyvinyl alcohol/sodium alginate (PVA/SA) hydrogel systems achieve 60.8 ± 3.7% encapsulation efficiency with zero-order release kinetics (r = 0.993), providing sustained compound delivery essential for immunomodulatory studies [194]. β-cyclodextrin complexation shows particular promise, with hydroxypropyl-β-cyclodextrin (HPβCD) achieving stability constants of 424 M^−1^ compared to 401 M^−1^ for methylated-β-cyclodextrin (MβCD), increasing water solubility of curcumin by 31-fold [195,196].

Liposomal encapsulation using soya lecithin demonstrates successful delivery of copper nanoparticles in cancer cell systems, achieving cytotoxic effects against MCF-7 breast cancer cells [19]. Mesoporous silica nanoparticles (MSNs) enable pH/glutathione-responsive release of poorly water-soluble compounds including quercetin, curcumin, and colchicine [197,198,199,200]. Amino-functionalized MCM-41 and KCC-1 variants conjugated with folic acid provide targeted delivery with controlled release profiles [201]. Recent studies demonstrate that surface-modified MSNs (acetylated, succinylated, polyethylene glycol (PEG)ylated) exhibit differential localization in 3D tumor microtissues, with acetylated MSNs showing superior tumor cell targeting in collagen-containing microenvironments, leading to significant reduction in tumor organoid size [202].

### 4.2. Standardization Requirements for Immune-Organoid Studies

#### 4.2.1. Protocol Harmonization

Significant international standardization efforts provide frameworks for organoid research. The Korean Organoid Standards Initiative, launched in September 2023 through partnership between the Ministry of Food and Drug Safety and Sungkyunkwan University [138], has published comprehensive guidelines across multiple organs (heart, liver, kidney, lung, brain, skin) with specific protocols for manufacturing, packaging, transport, and storage [138,142,143,144,203]. Chinese national standards include T/CMBA 017-2022 for gastrointestinal epithelial organoids and T/CSCB 0005-2022/0006-2022 for human intestinal and cancer organoids, which have demonstrably improved gastrointestinal tumor organoid construction success rates from 50% to 90% [141].

For natural product-organoid co-culture studies, specific standardization requirements emerge from recent methodological advances. Essential parameters include

- Immune cell ratios: 1:1 to 5:1 T cell:organoid ratios optimized for specific assay types, with 5:1 ratio specifically validated for cytotoxicity assays [134]
- Culture duration: Short-term exposure (24–48 h) for immediate cytotoxic responses versus extended culture (7–14 days) for T cell expansion and memory formation [134]
- Medium composition: Balanced mixtures of organoid and T cell media to maintain both populations’ viability [134]
- Natural product exposure protocols: Pre-treatment strategies demonstrate enhanced immunomodulatory effects in drug studies, including IFN-γ pre-treatment protocols enhancing T cell reactivity [134] and small molecule inhibitors improving chimeric antigen receptor T (CAR-T) cell efficacy in organoid models [204,205], though detailed evaluation of pre-treatment versus co-treatment timing for natural compounds specifically remains, to our knowledge, a significant research gap
- Natural product-specific considerations for immune co-cultures emerge from established protocols and compound characteristics:

1. Compound stability varies between culture media, as demonstrated by curcumin’s pH-dependent degradation [191,192] and the protective effects of serum proteins [193].
2. Optimization of exposure protocols follows principles established in organoid-T cell co-culture systems [134], with timing and sequential exposure requiring empirical determination.
3. Matrix penetration challenges, evident from the 6.6–31-fold higher concentrations required in 3D systems [95,178], are further complicated by immune cell migration and matrix remodeling.

The alignment of these international standards with natural product-specific requirements creates a foundation for reproducible immunomodulatory studies. That said, dedicated protocols addressing the unique physicochemical properties of natural products—particularly regarding compound stability, penetration kinetics, and batch standardization in 3D co-culture systems—require further development to enable reliable multi-center investigations.

#### 4.2.2. Quality Control Metrics

Validated high-throughput platforms demonstrate achievable standards. Van Hemelryk et al. established viability thresholds of 150% (day 10 vs. day 0) with coefficient of variation < 0.22 and Z-factor > 0.4 for prostate cancer organoid drug screening [206]. Du et al. achieved Z’-factor > 0.5 with signal-to-background ratios of 5–6 in miniaturized 3D organoid platforms [207].

Spatial uniformity substantially affects reproducibility. Boehnke et al. demonstrated that proper plate sealing reduces CV from 18.97% (unsealed) to 9.37% (sealed) in high-throughput organoid assays [208]. Passage consistency requires standardized split ratios (1:3 to 1:4) with quality assessment of marker expression and karyotype analysis every 5–10 passages [209,210].

#### 4.2.3. Reproducibility Challenges

Inter-laboratory variation remains substantial. Phipson et al. identified experimental batch as the strongest contributor to transcriptional variation in kidney organoids, exceeding biological differences [211]. Jensen and Little emphasize that organoids are not organs, highlighting sources of variation including matrix effects, passage number, and culture conditions [212].

Matrix variability presents particular challenges for natural product studies. Hughes et al. identified >1000 unique proteins in Matrigel with considerable batch-to-batch variability affecting compound diffusion and bioactivity [213]. Alternative approaches using tissue-derived extracellular matrix hydrogels show promise for reducing variability while maintaining physiological relevance [214].

Compound preparation standardization is essential. Verduin et al. note that drug compounds require experimental optimization due to the variable nature of organoids, with natural products presenting additional challenges due to their complex composition and stability issues [215]. Implementation of standardized protocols could significantly reduce inter-laboratory variation, enabling reliable multi-center studies of immunomodulatory natural products.

**Figure 3:**
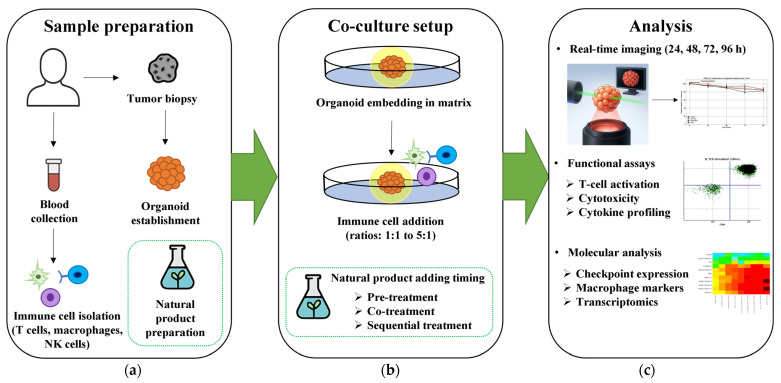
Proposed workflow for natural product immunomodulation testing in cancer organoid-immune co-culture systems. (**a**) Sample preparation involves patient tumor biopsy for organoid establishment, peripheral blood collection for immune cell isolation (T cells, macrophages, NK cells), and standardized natural product preparation. (**b**) Co-culture setup demonstrates organoid embedding in matrix followed by immune cell addition at optimal ratios (1:1 to 5:1), with three alternative natural product treatment strategies: pre-treatment, co-treatment, or sequential treatment protocols. (**c**) Multi-parametric analysis endpoints include real-time imaging monitoring organoid viability (24–96 h), functional assays measuring T cell activation (CD69/CD25 expression) and cytotoxicity, and molecular profiling using heatmap analysis of cytokine production. Representative/illustrative data shown demonstrate the feasibility of this comprehensive workflow for systematic evaluation of natural product immunomodulation in physiologically relevant 3D systems. 3D, three-dimensional; CD25, interleukin-2 receptor alpha chain; CD69, cluster of differentiation 69; h, hours; NK, natural killer.

These reproducibility challenges are particularly important for immunomodulatory studies, where batch-to-batch variations in both natural products and immune cell preparations could significantly impact outcomes. Long-term co-culture studies face additional challenges including immune cell exhaustion, phenotype drift, and potential loss of cytotoxic function over extended periods. These limitations necessitate careful temporal monitoring of immune cell markers and functional validation throughout experimental timelines using comprehensive workflows designed for organoid-immune co-culture systems (Figure 3).

### 4.3. Technological Convergence Enabling Natural Product Discovery

The complete absence of natural product immunomodulation studies in organoid-immune co-cultures, as documented in Section 3, coincides with remarkable technological capabilities that now make such investigations feasible.

#### 4.3.1. Spatial Multi-Omics Integration

Recent advances in spatial transcriptomics offer unparalleled opportunities for understanding natural product mechanisms in organoids. Even as platforms achieving 2-μm resolution were announced for commercial launch in 2024 [216], current technologies already demonstrate remarkable capabilities in mapping compound distribution and cellular responses. The foundational spatial transcriptomics platform operates at 100-μm resolution [217], while newer methods including Slide-seq (10-μm) [218] and MERFISH (single-molecule resolution) [219] enable increasingly precise spatial analysis. Wahle et al. successfully combined multiplexed protein mapping (53 antibodies across 21 imaging cycles), single-cell transcriptomics (212,781 cells), and chromatin accessibility analysis (151,684 cells) across retinal organoid development, providing a framework for studying natural product effects at multiple molecular levels [220]. The application of optogenetics with spatial transcriptomics, as demonstrated by Legnini et al., enables controlled perturbation studies using light-inducible systems (single-cell perturbation transcriptomic screen (SCPTS), photoactivatable Tet-On system (PA-TetON), photoactivatable Cre-Lox system (PA-Cre-Lox)) combined with both 10× Visium and Molecular Cartography platforms that could elucidate natural product mechanisms with exceptional precision [221].

Even so, natural products present unique analytical challenges. Their autofluorescence often interferes with imaging modalities, particularly for flavonoids and alkaloids that exhibit intrinsic fluorescence at wavelengths overlapping with common fluorophores [222,223]. Complex natural product mixtures containing thousands of structurally diverse molecules further complicate molecular tracking, with matrix effects and ion suppression creating additional barriers for accurate spatial quantification [224,225]. Cell2location and similar Bayesian models show promise for deconvoluting these complex signals, having demonstrated high-sensitivity spatial mapping with superior performance (root mean square error, root mean square error (RMSE) 0.0373) in various tissue types including brain, lymph node, and gut tissues [226]. Integration costs remain substantial, with commercial platforms requiring $11,000–14,000 per slide [227], creating barriers to widespread adoption. Conversely, recent advances in automation demonstrate 35% reduction in sample preparation time [228], while comprehensive reviews project continued improvements in efficiency and accessibility through technological maturation [229].

#### 4.3.2. Machine Learning for Response Prediction

AI models have achieved remarkable accuracy in predicting drug responses in organoid systems. Kong et al. developed a network-based machine learning approach that predicted anti-cancer drug efficacy with R^2^ = 0.98 for colorectal cancer and R^2^ = 0.89 for bladder cancer across 114 colorectal and 77 bladder cancer patients [230]. Importantly, their model demonstrated clinical relevance with predicted responders showing significantly better survival outcomes (*p* = 0.014 for colorectal, *p* = 0.01 for bladder cancer) [230]. Recent advances include Cellos, which employs deep learning with StarDist-3D using a ResNet backbone for quantitative 3D organoid analysis, enabling high-throughput deconvolution of organoid dynamics at cellular resolution from approximately 100,000 organoids containing 2.35 million cells [231].

For natural products, specialized approaches are emerging. The YOLOv8-based Deliod model achieves 87.5% mean average precision at 50% Intersection over Union (IoU) (mAP50) accuracy for intestinal organoid detection, demonstrating the feasibility of automated screening [232]. OrganoIDNet specifically addresses organoid-immune cell interactions, providing automated analysis of pancreatic ductal adenocarcinoma (PDAC) organoid-peripheral blood mononuclear cell (PBMC) co-cultures from time-resolved imaging data—essential for immunomodulatory natural product assessment [233]. This platform categorizes organoids by size, health status, and eccentricity while tracking longitudinal responses to chemotherapy and immunotherapy over 100 h periods [233].

Nonetheless, given these advances, natural products present unique challenges: chemical complexity requiring expanded descriptor sets, limited training data, and batch-to-batch variability affecting model reliability. Realistic near-term applications include structure-activity relationship modeling for compound prioritization and automated quality control for standardization.

#### 4.3.3. Organ-on-Chip Integration

Microfluidic platforms offer solutions to natural product delivery challenges in organoid systems. Schuster et al. developed an automated platform capable of testing 200 organoids simultaneously with real-time analysis, achieving 20 independent fluidic conditions through a gravity-driven flow system [234]. Recent innovations include perfusable vascular networks that maintain functionality for up to 30 days, as demonstrated by Quintard et al., who achieved long-term perfusion at 1 μL/min in their 10-channel cyclic olefin copolymer (COC) system [235].

Technical specifications have been optimized for organoid culture: flow velocities of 100–7500 μm/s enable proper nutrient delivery and waste removal, with physiological shear stress values of 1–10 dynes/cm^2^ mimicking tumor-vascular interactions [236]. Although mechanical stimulation has been shown to enhance organoid maturation in various systems, specific parameters for natural product testing in mechanically stimulated organoids require further investigation.

Additionally, natural products pose specific challenges for microfluidic systems. The general review by Dressaire and Sauret highlights clogging as a major challenge in microfluidic systems operating at low Reynolds numbers [237]. Hydrophobic compounds present absorption challenges in polydimethylsiloxane (PDMS) devices, with small molecules showing >70% loss within 3 h [238]. Surface modifications using PEG-based coatings reduce PDMS contact angles from 108° ± 5° to 52° ± 3°, significantly improving hydrophilicity and reducing nonspecific absorption [239].

Recent advances integrate immune components directly into organ-on-chip systems. Kroll et al. developed millifluidic chips enabling PBMC circulation through kidney organoids for T cell bispecific antibody assessment [149]. These platforms enable in situ monitoring of immune-tumor interactions through transparent observation windows, allowing real-time confocal microscopy of natural product effects. Emerging organ-on-chip technologies now enable evaluation of natural product immunomodulation in physiologically relevant microenvironments with integrated immune cell populations, bridging the gap between traditional 2D cultures and clinical outcomes.

### 4.4. Clinical Translation Pathway

The merger of validated organoid-immune co-culture platforms with advanced analytical technologies creates immediate opportunities for translating natural product immunomodulation discoveries into clinical applications.

#### 4.4.1. Regulatory Considerations

The FDA Modernization Act 2.0, signed into law 29 December 2022, authorizes “cell-based assays” and “microphysiological systems” as alternatives to animal testing, which the FDA and scientific community interpret to include organoid technologies [240,241].

Natural product-organoid combinations face unique regulatory considerations. Quality control requirements are complicated by the lack of defined active pharmaceutical ingredients in natural products. Organoid-based potency assays could establish biological standardization, complementing chemical analysis. The combination of organ-on-chip technology with pharmacokinetic modeling shows promise as a substitute for traditional animal studies, with the FDA aiming to make animal testing “the exception rather than the norm” within 3–5 years [242].

Clinical validation pathways are emerging through studies like Narasimhan et al., which demonstrated successful PDO generation in 68% (19/28) of colorectal cancer patients with peritoneal metastases, with one patient showing partial response to PDO-guided gemcitabine-capecitabine therapy [243]. However, natural product trials require modified endpoints acknowledging longer immunomodulation timelines compared to cytotoxic agents.

#### 4.4.2. Economic Realities

Current cost structures present implementation barriers. Whereas comprehensive precision medicine platforms including genomic and preclinical testing cost $12,743–21,769 per patient (in 2021 Australian dollars) in clinical implementation [244], specific organoid-related cost data remains limited. Clinical implementation studies demonstrate 68.8% success rate for completing organoid drug testing within 7 weeks in metastatic colorectal cancer [245]. This technological advancement, combined with high predictive accuracy (as exemplified by Vlachogiannis et al.’s finding of 100% sensitivity and 93% specificity in predicting treatment responses for 15 patients from their 71-patient cohort) [14], demonstrates the potential of organoids to transform drug development by enabling accurate patient-specific treatment selection.

Traditional drug development averages $1.3–2.87 billion per approved drug, with median research and development (R&D) costs of $985 million and mean costs of $1.336 billion (2018 US dollars) [246,247]. Given that 90% of drugs fail in clinical trials, with lack of efficacy accounting for 40–50% of failures [97], organoids’ high predictive accuracy offers significant economic advantages.

For clinical viability, costs must decrease while maintaining quality. Automated organoid platforms achieve coefficient of variation of 3.56% (range 2.2–5.6%), representing up to 5–10-fold improvement in reproducibility compared to other organoid systems [145]. Cost-reduction strategies such as L-WRN conditioned medium show promise [248]. Insurance coverage remains a major barrier, with studies showing only 50% or less of personalized medicine tests covered by insurers due to lack of clinical utility evidence [249].

#### 4.4.3. Integration with Standard Care

Realistic implementation requires phased approaches. Multiple clinical trials demonstrate organoid utility in treatment selection. The CinClare trial achieved 84.43% accuracy in predicting chemoradiation response in locally advanced rectal cancer patients (n = 80) [250]. For metastatic gastrointestinal cancers, PDOs demonstrated 100% sensitivity and 93% specificity in predicting treatment responses across patients in phase I/II clinical trials [14].

The Drug Efficacy Testing in 3D Cultures (DET3CT) platform represents current best practices for ovarian cancer, achieving 6-day turnaround for drug sensitivity profiling with >90% success rate [251]. Alternative approaches include micro-organospheres that enable drug testing within 14 days for colorectal and lung cancers [252]. Molecular tumor boards have been developed to integrate genomic data for clinical decision support, with digital infrastructure platforms like the Molecular Tumor Board Portal enabling automated reporting and virtual collaboration for precision oncology [253].

### 4.5. Priority Research Directions

Based on current evidence and technological capabilities, immediate priorities include:

1. Systematic screening: Leverage high-throughput platforms to test natural products specifically in organoid-immune co-cultures, prioritizing compounds with established immunomodulatory effects in 2D systems.
2. Mechanistic validation: Apply spatial transcriptomics and live imaging to understand how natural products modulate tumor–immune cell interactions in 3D, not just direct cytotoxicity.
3. Formulation optimization: Develop organoid-specific delivery systems utilizing tumor-penetrating peptides and other innovations. Recent advances with internalizing RGD (Arg-Gly-Asp) peptide (iRGD) demonstrate enhanced drug delivery in 3D tumor spheroids [254], providing a framework for natural product optimization.
4. Clinical correlation: Establish prospective trials comparing organoid predictions with patient responses, following successful frameworks from colorectal and pancreatic cancer studies.
5. Standardization: Implement quality control measures achieving coefficient of variation < 15%, as demonstrated in automated screening platforms. Renner et al. [145] achieved average CV of 3.56% within batch for organoid size distribution, while Boehnke et al. [208] demonstrated CV of 9.37% with proper sealing membranes in high-throughput organoid assays.

### 4.6. Realistic Outlook

The union of natural products, immunotherapy, and organoid technology offers genuine potential for advancing cancer treatment. Technical barriers are being systematically addressed through platform innovations and standardization efforts. Clinical validation is progressing through multiple prospective trials demonstrating strong organoid-patient correlations, with studies achieving 93–100% sensitivity and specificity for treatment response prediction [14,132,250].

Success depends on avoiding previous pitfalls of natural product research—overpromising based on limited data and focusing solely on cytotoxicity. By systematically investigating immunomodulatory mechanisms in physiologically relevant organoid-immune co-cultures, the field can deliver meaningful clinical advances. Acknowledging that this review highlights promising opportunities, not all natural products will successfully translate to organoid-immune co-culture platforms. Compounds with extreme hydrophobicity, pH sensitivity, or those requiring concentrations toxic to immune cells may prove unsuitable. Future studies should establish clear selection criteria based on physicochemical properties and preliminary toxicity profiles.

## 5. Conclusions

This review reveals a striking paradox: while natural products demonstrate extensive immunomodulatory potential and organoid technology offers innovative platforms for studying tumor–immune interactions, no published studies have investigated natural product-mediated immunomodulation in organoid-immune co-culture systems. This research gap, despite validated platforms and strong rationale, represents both a significant void and an immediate opportunity.

Key priorities for advancing the field:

1. Systematic screening of immunomodulatory natural products in organoid-immune co-cultures—Research teams should prioritize compounds with established 2D immunomodulatory evidence (curcumin, EGCG, resveratrol, medicinal mushroom polysaccharides) using standardized protocols: 1:1 to 5:1 immune:organoid ratios [134], 7–14 day culture periods, and multi-parametric readouts including checkpoint expression, cytokine profiles, and functional cytotoxicity.
2. Development of natural product-specific delivery systems—Bioengineers must address the unique challenges of hydrophobic compounds (curcumin solubility < 11 ng/mL) [188] and high molecular weight polysaccharides (lentinan 400–800 kDa) [52] through innovations in nanoparticle formulations, cyclodextrin complexation, and matrix-penetrating peptides optimized for 3D systems.
3. Clinical validation through prospective trials—Oncologists should integrate organoid-based natural product screening into clinical workflows, following the successful framework of the TUMOROID [133] and CinClare trials [250], with endpoints specifically designed for immunomodulatory effects rather than direct cytotoxicity alone.

The path forward is clear: The coming together of validated organoid-immune co-culture platforms (achieving 81–84% clinical correlation in pooled analyses) [146], FDA regulatory support through the Modernization Act 2.0 [17], and millennia of natural product wisdom creates a transformative opportunity. Success requires moving beyond cytotoxicity-focused studies to systematically investigate immunomodulatory mechanisms in physiologically relevant 3D systems. With organoid establishment now routine (68–90% success rates) [14,132,148] and standardized protocols emerging globally [138,140,141,142,143,144], the field stands ready to unlock the therapeutic potential of natural product immunomodulation for precision cancer therapy.

## Figures and Tables

**Table 1:** Comparison of research platforms for natural product evaluation in cancer models.

Platform Type	Key Features	ImmuneIntegration	Natural ProductImmunomodulation Studies
2D Culture	Simple, high-throughput	Artificial co-culture only	Extensive
Patient-Derived Organoids	50–90% success rate [14,132,148]	No	None (cytotoxicity only)
PDO + ALI Co-culture	33–50% T cell generation [134,135]	Yes (T, B, NK, macrophages) [8]	None identified
Organ-on-Chip	Perfusion capability	Yes [149]	None identified

Abbreviations: 2D, two-dimensional; PDO, patient-derived organoid; ALI, air-liquid interface; NK, natural killer. Note: Success rates for patient-derived organoids vary by cancer type and study.

**Table 2:** Natural products tested in patient-derived cancer organoid systems.

**Compound**	**Cancer Type**	**Organoid Model**	**Effect Measured**	**Key Findings**	**Reference**
Luteolin	Gastric	PDO	Cytotoxicity	IC50: 27.19 μM, superior to carboplatin (37.87 μM)	[150]
			Gene expression	1059 DEGs (785 up, 274 down)	
			Pathway analysis	FOXO, MAPK, NF-κB pathway modulation	
Curcumin	Colorectal	PDO	Cytotoxicity	IC50: 20–50 μM across multiple PDOs	[151]
			Metabolic response	Altered phenylalanine/tyrosine/tryptophan biosynthesis	
			Metabolomics	NAD+ pathway and purine metabolism changes	
Resveratrol	Breast	PDO	Cytotoxicity	79.2% response rate (19/24), >50% cell death at 100 μM, 96 h	[16]
			Proliferation	Reduced EdU incorporation in sensitive organoids	
			Oxidative stress	ROS elevation in sensitive vs. resistant organoids	
			STAT3 signaling	pSTAT3 nuclear translocation: 89.47% vs. 20%	
Resveratrol	Bladder	PDO	Cytotoxicity	55.56% sensitivity (10/18 organoids) at 100 μM, 96 h	[152]
			Ferroptosis	Ferroptosis contribution: 22.57% (ferrostatin-1 rescue)	
			ROS generation	Peak ROS at 12 h (*p* < 0.0001)	
			Cell death pathways	GPX4/xCT downregulation, multi-modal cell death	
Quercetin	Breast	PDO	Cytotoxicity	IC50: 7.56–21.77 μM across 4 PDOs	[153]
			Chemosensitization	Enhanced sensitivity to docetaxel, epirubicin, cisplatin at 10 μM	
			Drug synergy	Synergistic effects (CI < 1)	
Manoalide	Lung	3D organoid	EGFR-TKI sensitization	10–15 μM overcomes osimertinib resistance	[154]
			Ferroptosis	Mitochondrial Ca^2+^ overload-induced ferroptosis	
			Metabolism	4-fold succinate increase	
			Signaling pathways	KRAS-ERK inhibition (ROS-dependent)	
Chaga mushroom (*Inonotus obliquus*) extract	Bladder (canine)	Organoid	Cytotoxicity	Dose-dependent inhibition (25–100 μg/mL)	[155]
			Cell cycle	G0/G1 arrest, ↓Cyclin A2, D1, E1	
			Apoptosis	P-ERK inhibition (6–48 h, *p* < 0.05)	
			Stemness markers	↓CD44, SOX2, YAP1 expression	

Abbreviations: PDO, patient-derived organoid; IC50, half-maximal inhibitory concentration; EGFR-TKI, epidermal growth factor receptor-tyrosine kinase inhibitor; DEGs, differentially expressed genes; ROS, reactive oxygen species; pSTAT3, phosphorylated STAT3; CI, combination index; ERK, extracellular signal-regulated kinase; FOXO, forkhead box O; MAPK, mitogen-activated protein kinase; NF-κB, nuclear factor kappa B; NAD+, nicotinamide adenine dinucleotide; GPX4, glutathione peroxidase 4; xCT, cystine/glutamate transporter; KRAS, Kirsten rat sarcoma viral oncogene homolog; SOX2, SRY-box transcription factor 2; YAP1, Yes-associated protein 1; P-ERK, phosphorylated ERK. Symbols: ↓ indicates decreased or downregulated expression.

## Data Availability

Data are contained within the article.
